# Does metabolic syndrome predict surgical complications? A protocol for a systematic review and meta-analysis

**DOI:** 10.1186/s13643-017-0515-6

**Published:** 2017-06-17

**Authors:** Philip Norris, Nicholas Ralph, Clint Moloney

**Affiliations:** 1 School of Nursing & Midwifery, University of Southern Queensland, Toowoomba, Australia; 2 Institute of Resilient Regions, University of Southern Queensland, Toowoomba, QLD 4370 Australia

**Keywords:** Metabolic syndrome, Surgery, Safety, Risk, Prevalence, Complications, Adverse events

## Abstract

**Background:**

Metabolic syndrome (MetS) is defined by an accumulation of risk factors that include cardiovascular disease, diabetes, chronic high blood pressure, obesity, and hypercholesterolaemia which results in an increased risk of developing serious chronic diseases. MetS is widespread as it is estimated to affect up to 30% of the global population. For people with MetS who undergo surgery, an emerging body of literature points to significantly poorer postoperative outcomes compared with non-affected populations. Surgical patients with MetS are at significantly higher risk of a range of adverse outcomes including death, morbid cardiovascular events, coma, stroke, renal failure, myocardial infarction, and surgical site infections. Increased complication rates result in prolonged hospital stays, a greater need for post-hospitalisation care, and reduced effectiveness of surgical interventions.

**Methods/design:**

We will search the following electronic bibliographic databases: MEDLINE, EMBASE, ScienceDirect, and CINAHL, and the reference lists of included articles. We will also search for unpublished literature. Two authors will screen titles and abstract information independently and select studies according to established inclusion and exclusion criteria. Data will be extracted by the study investigators using Review Manager 5 and will include information on demographics, incidence, prevalence, and outcome variables. Subgroup analysis and sensitivity analysis will be performed to assess the heterogeneity of included studies. Meta-analysis will also be carried out if appropriate study groups are identified. A descriptive narrative for statistical data will also be provided to highlight findings of the systematic review and meta-analysis.

**Discussion:**

This study will report and summarise adverse outcomes among adult patients with MetS undergoing surgery across a range of surgical specialties. Developing insights into outcomes of this population of interest is necessary to develop guidelines towards better management of surgical patients with metabolic syndrome.

**Systematic review registration:**

PROSPERO CRD42016051071

**Electronic supplementary material:**

The online version of this article (doi:10.1186/s13643-017-0515-6) contains supplementary material, which is available to authorized users.

## Background

Metabolic syndrome (MetS) is a widespread health concern which leads to the development of cardiovascular disease, diabetes, chronic high blood pressure, obesity, and hypercholesterolaemia [1]. Individuals with MetS typically display symptoms of hypertension, increased fasting glucose, elevated triglycerides, obesity (either using BMI or waist circumference), and decreased high-density lipoprotein concentrations [2]. The presence of any three out of five of these symptoms or risk factors constitutes a diagnosis of MetS [3]. There are several definitions for MetS, and while they share the same overarching risk factors, diagnostic criteria slightly differ across each definition. The World Health Organization, a Joint Interim Statement (JIS) produced by leading health organisations, and the National Cholesterol Education Program Adult Treatment Panel III have produced the most commonly accepted definitions of MetS [1, 3, 4]. Using these definitions, an estimated 35 to 40% of the population in developed countries have MetS [5]. Having MetS increases the risk of developing type 2 diabetes mellitus fivefold and cardiovascular disease threefold [6]. It is also associated with the occurrence of a wide range of cancers including colon, pancreatic, liver, and breast cancer [2].

Complications can be defined as any deviation from the normal surgical pathway and can occur intraoperatively or postoperatively [7]. Having MetS increases the number of complications during and after surgery when compared to unaffected populations. Commonly reported adverse events seem to indicate that patients with MetS experience higher rates of mortality, increased instances of postoperative morbidity including cardiovascular complications, and slower recovery of function across both cardiac and non-cardiac surgery [8–11]. Non-routine discharge, protracted length of stay, and increased costs associated with hospitalisation are also detailed in the literature [12–14].

Increased surgical mortality is reported among patients with MetS. In a study of 310,208 patients with MetS undergoing non-cardiac surgery, a twofold increased risk of death was observed when comparing with patients without MetS [8]. Mortality is also reported as a risk among patients with MetS who undergo renal transplant surgery [15]. In cardiac surgery, having MetS increases the risk of mortality in patients undergoing coronary artery bypass grafting (CABG), with women particularly affected [10, 16–19], although one study of 100 patients did not detect an increase in the prevalence of mortality after CABG surgery [20]. In a study reporting on outcomes among 1222 patients undergoing aortic and mitral valve-replacement surgery, significantly higher rates of mortality were reported by the authors [21]. MetS also appears to prognosticate mortality after gastrectomy for gastric cancer as higher mortality was detected [12].

In addition to increased risks of mortality, patients with MetS appear to be at risk of perioperative morbidity with varied complications reported across a range of surgical procedures. For example, patients undergoing total joint arthroplasty (TJA) with MetS were reported to experience higher overall complication rates of 49, 8, and 8% for uncontrolled MetS, controlled MetS, and no MetS, respectively [11]. Similar studies reported higher overall complication rates in comparable patient groups who underwent TJA surgery [22–26]. In one retrospective cohort study of 1553 patients over 7 years, MetS was identified as a significant risk factor for deep vein thrombosis after total joint arthroplasty [27]. Even in isolated ankle fracture surgery, an increased risk of complications and slower return to independent functional mobility were reported among patients with MetS [13]. Several complications were documented in patients who underwent cardiovascular surgery including poorer patency of vascular grafts, bleeding, dysrhythmia, atelectasis, deep vein thrombosis, stroke, myocardial infarction, and infection [10, 14, 20, 26, 28, 29].

Postoperative complications are associated with slower recovery of function, poorer prognosis, extended length of stay, and discharge to non-routine environments. For example, the effectiveness of surgical interventions is reduced in the presence of MetS with these patients experiencing far poorer long-term surgical and functional outcomes after CABG surgery [18]. Patients with MetS are more likely to experience protracted lengths of stay in the hospital after CABG surgery [14], acute orthopaedic trauma [13], spinal fusion surgery [30], total joint arthroplasty [11], and gastric bypass surgery [31]. However, no statistically significant difference in the length of stay between patients with or without MetS has also been reported in several studies [20, 22, 23]. Patients with MetS are also more frequently discharged to non-routine environments (to an acute or subacute rehabilitation centre, skilled nursing facilities, and other institutions) [26]. Patients with MetS also experience higher readmission rates in the first 12 months following liver transplantation [32] and CABG surgery [14]. As a result, costs associated with treating surgical patients with MetS are significantly higher across a range of surgical types [12, 30, 33].

Considering the wealth of literature on the topic and the absence of systematic reviews in the area, this review is both timely and needed. This situation demands the undertaking of a systematic review of surgical patients with MetS to articulate risks as well as the development of a care pathway to better manage these risks. Further knowledge about the nature and prevalence of complications from surgery related to MetS is needed to help formulate targeted interventional research studies aimed at improving surgical outcomes among patients with the condition. Collating this data is also vital for observing and recording trends in MetS and surgical outcomes and to contribute to the design of further prevalence studies or interventional research.

## Objectives

The objective of this review is as follows:

- To evaluate the effect of metabolic syndrome on the occurrence of surgical complications in adult surgical patients (18 years or older).

## Methods

### Protocol

Recommendations from the Preferred Reporting Items for Systematic Reviews and Meta-Analyses Protocols (PRISMA-P) 2015 statements have been used to develop the methods for undertaking this systematic review [34]. The systematic review protocol has been registered in the International Prospective Register of Systematic Reviews (PROSPERO): 41427. A PRISMA-P file is attached (see Additional file 1)

## Eligibility criteria

### Population

Adult surgical patients 18 years of age and older with metabolic syndrome will be included in the review of studies.

### Outcome

The primary outcome will be the risk of surgical complications in patients with MetS at various time intervals. We will report risk as incidence rate, incidence proportion, odds, point prevalence, or period prevalence. Intervals are defined as follows:

- Intraoperative period (during surgery)
- Up to 30 days after surgery as short-term
- >26 weeks as long-term

Surgical complications will be broadly defined as any deviation from the normal surgical pathway and included in a summary presentation of findings. We will primarily address:

- Mortality during surgery and up to 30 days postoperatively
- Surgical site infection (SSI)
- Cardiovascular complications (e.g. stroke, arrhythmia, myocardial infarction)

We will record the occurrence of postoperative SSI as defined by the CDC criteria [35] or the authors’ definition of SSI. We will not differentiate the depth of infection. We will document sepsis or septic shock under this outcome.

We will also record secondary outcomes as follows:

- Readmission within 30 days
- Mortality within 12 months
- Length of stay
- Cost
- Resource usage (as defined by authors)

### Study design

Studies will be restricted on the basis of design. Only retrospective or prospective observational studies, cross-sectional studies, cohort studies, case-control studies, and systematic reviews/meta-analyses will be included. While the authors are not aware of randomised controlled trials which test interventions for surgical patients with MetS, this study type will be excluded on the basis of methodological inappropriateness for answering the research question posed by the protocol.

## Search strategy

We will search the following databases: MEDLINE, EMBASE, ScienceDirect, and Cumulative Index to Nursing and Allied Health Literature (CINAHL), and the reference lists of included articles. Search terms will reflect various names for MetS such as “metabolic syndrome”, “syndrome x”, and the “deadly quartet” while a range of synonyms for “surgery” will be included. The full search strategy will include only terms relating to or describing the phenomena of interest and is described in the Appendix. The search strategy has been peer reviewed by a research librarian experienced in systematic review search strategies. All imported data from the search strategies will be stored in EndNote (ThomsonReuters) [36]. Duplicates will be removed from the EndNote library. Data extraction will be managed within Review Manager 5 (Cochrane) on institutional licence to the institution of the lead author.

Searches will be limited to peer-reviewed full-text articles in the English language, and letters, abstracts, and editorials are to be excluded. There will be no geographical limitation on the included studies. No date limitations will be placed on the search strategy. The searches will be re-run immediately prior to the final analyses and further studies retrieved for inclusion as appropriate. Where necessary, authors of studies will be contacted for further information. Inclusion and exclusion criteria will be employed rigorously and are presented in Table 1.

**Table 1:** Example inclusion and exclusion criteria

Example inclusion criteria	
□ Observational studies (e.g. cohort study)	□ Diagnosed with metabolic syndrome with ≥3 risk factors
□ Adult human patients (18 years or older)	□ Published peer-reviewed articles
□ Undergoing surgery	
Example exclusion criteria	
Publication type	Study design
□ Narrative reviews	□ Interventional studies
□ Editorials	Study population
□ Government reports	□ Animals
□ Books or book chapters	□ Children
□ Conference proceedings	□ Pregnant women
□ Commentaries	Study procedure
□ Consensus statements	□ Minor procedures (e.g. lesion removal, cystoscopy, endoscopy)
□ Clinical guidelines	□ Cardiac catheterisation
□ Lectures and presentations	

## Selection of studies

Two reviewers (PN and NR) will independently screen titles and abstracts against inclusion and exclusion criteria to identify potential studies for review. Following the initial search for studies, we will retrieve full-text copies of articles which are judged as potentially relevant. Two reviewers (PN and NR) will independently assess full-text articles retrieved for compliance with inclusion and exclusion criteria. As the criteria for defining MetS may vary, we will accept definitions of MetS as used by the study authors. Where required and possible, we will contact study authors where it is unclear if the study meets inclusion or exclusion criteria. Any discrepancies in studies selected for inclusion in the review will be marked within data management software and resolved through discussion with a third reviewer (CM). Studies which appeared to be candidates for inclusion but excluded at this stage of review will be detailed in a table entitled “Characteristics of Excluded Studies” where a justification for exclusion will be listed. The final list of studies included in the review will be verified by all three reviewers, and a PRISMA flow chart will be provided detailing the steps taken in the full systematic review.

## Data extraction and management

Data will be extracted on the following and entered into Review Manager 5 to ensure the consistency of information retrieved and presented across studies [37]:

1. Study details: title, journal, author, year, city, and country where research was conducted, type of publication, and funding source
2. Methods: eligibility of study (as per inclusion criteria), study aim, data collection method, recruitment, and sampling methods
3. Participant demographics: number of participants, population demographics, MetS diagnostic criteria applied, reported complications
4. Outcome measures: estimates of, and data for, point and period prevalence, cumulative incidence, and incidence rate of surgical complications in adult surgical patients with MetS
5. Limitations: study biases as assessed by the Risk of Bias in Non-randomised Studies of Interventions (ROBINS-I) assessment tool used and limitations identified by study authors

## Risk of bias

The risk of bias will be assessed for included studies using the ROBINS-I tool specifically designed for appraising the quality of studies including conducting quality appraisal of non-randomised studies in systematic reviews [38]. Two reviewers (PN and NR) will appraise studies using the tool with the third reviewer (CM) to resolve discrepancies. The instrument can be employed for different study designs as a mechanism to appraise studies for internal and external validity relevant to assessing prevalence data. The instrument assesses representativeness, recruitment, sample size, reporting, data coverage, condition reliability, statistical analysis, and confounding factors using a simple “yes”, “no”, “unclear”, or “not/applicable”. Results of the assessment will be presented in table format.

We will also perform symmetry of funnel plots and Egger’s test to identify publication and selective reporting bias [39]. We will consider a *p* value <0.10 to be a statistically significant indicator of publication bias.

## Data collection and analysis

We will follow meta-analysis of observational studies in epidemiology (MOOSE) guidelines in the conduct of this review [40]. Data extracted from selected studies for inclusion will be presented in evidence tables. Descriptive narrative will accompany meta-analysis to summarise the prevalence of outcomes of interest for adult surgical patients with MetS according to age, surgical type, and healthcare setting (e.g. hospital, clinic) and any specific population demographics at risk of developing complications (e.g. men >65 years of age). Meta-analysis of outcome variables will be conducted where appropriate in reporting outcome estimates. The outcome variables will be grouped to identify similar patient populations and enable meta-analyses where the designs of studies are similar. Incidence and prevalence data will be presented with corresponding standard error and 95% confidence intervals using the exact binomial method to model variability of outcome frequency. The exact binomial method produces an exact confidence interval that is specifically informed by binomial distribution instead of an approximation to the binomial distribution. We will also use Poisson distribution where summary data involves counts of cases and the person-time of follow-up for each subgroup of interest.

We will meta-analyse data from comparable studies (where similar measures of frequency and outcome are reported) if two studies or more are available by outcome of interest. We anticipate that study and patient characteristics will differ across trials as they will likely use different measures of outcomes across a range of frequencies. We will therefore apply a random-effect model to incorporate anticipated heterogeneity in the analysis of data [21].

We will pool study-specific estimates depending on whether it is continuous or dichotomous data through a random-effect meta-analysis model. For continuous data, we will use the inverse variance approach with random-effect models to pool the standardised mean difference if studies employed different measures to calculate the same outcome or mean difference if studies used identical measures. For dichotomous variables, we will combine individual study data using the Mantel-Haenszel method. We will pool and report effect sizes as well as their 95% confidence limits. If quantitative data cannot be synthesised, we will use summary tables to describe the MetS definition, relevant comparator, and outcome of interest. Where studies have a minimal or major effect on overall estimates, we will moderate the variance of study-specific data using the Freeman-Tukey test. Included studies will be assessed for heterogeneity related to both methodological and clinical characteristics using Cochrane’s *Q* test [23]. In the presence of heterogeneity, subgroup analyses (e.g. age, sex, setting, and clinical outcome) will be performed and univariate meta-regression (*p* value <0.10 will be considered significant given the low power of these tests) will be carried out in order to estimate the effect of study-level covariates on the estimates of incidence and prevalence according to surgical specialty [25].

Sensitivity analyses will be conducted to identify the study-level factors that best describe the occurrence of complication. We will also conduct outlier analyses to determine the effects of certain studies on the pooled estimates of surgically related outcomes among the population of interest [26]. These analyses will indicate how estimated parameters of a pooled analysis differ if outlier studies are disregarded. Excluding such studies from a random-effect analysis will be performed and reported on.

## Discussion

This systematic review and meta-analysis is the first of its kind in addressing surgical complications likely associated with MetS. We aim to identify and report the rates of complications among surgical patients with MetS, specifically related to mortality, morbidity, length of stay, and 30-day readmission. Population demographics associated with the manifestation of identified risks will be reported and include findings which will help increase awareness and guide decision-making to improve the quality of care among the population of interest. As no other reviews appear to exist which specifically address the research question posed for this review, it is anticipated that comparisons will not be made with similar publications. Finally, conclusions which draw on highlighted estimations of prevalence and incidence will be provided in this review. The limitations of this systematic review will be detailed and discussed. Suggestions for future avenues of research will be provided to encourage clinical researchers to give attention to reducing risks faced by surgical patients with MetS.

The authors are not aware of any guidelines developed which target adult surgical patients with MetS. The findings of the review will be used to develop criteria for the initial round of a Delphi study in which a multi-disciplinary cross section of experts in MetS and surgery will be invited to develop clinical guidelines for the management of surgical patients with MetS. The efficacy of these guidelines in reducing complications will be tested as part of a broader study investigating patients with metabolic syndrome undergoing surgery.

## Appendix

**Table 2:** Example search strategies

Search date	MEDLINE search strategy
December 12, 2016	(((metabolic syndrome[Title/Abstract] OR syndrome x[Title/Abstract]) OR “metsy”[Title/Abstract]) OR “deadly quartet”[Title/Abstract]) AND (((((((((((surgery[Title/Abstract] OR “surgeries”[Title/Abstract]) OR “operation”[Title/Abstract]) OR “operative”[Title/Abstract]) OR “intervention”[Title/Abstract]) OR “interventions”[Title/Abstract]) OR “operations”[Title/Abstract]) OR “perioperative”[Title/Abstract]) OR “intraoperative”[Title/Abstract]) OR “preoperative”[Title/Abstract]) OR “postoperative”[Title/Abstract]) OR “surgical”[Title/Abstract]) AND (“1998/01/01”[PDAT] : “2017/12/31”[PDAT]) AND (“1998/01/01”[PDAT] : “2017/12/31”[PDAT])
	CINAHL search strategy
December 12, 2016	(((TI metabolic syndrome) OR (AB metabolic syndrome)) OR ((TI syndrome x) OR (AB syndrome x)) OR ((TI Metsy) OR (AB Metsy)) OR ((TI Metsyn) OR (AB Metsyn)) OR ((TI Deadly Quartet) OR (AB Deadly Quartet))) AND (((TI intervention) OR (AB intervention)) OR ((TI interventions) OR (AB interventions)) OR ((TI operation) OR (AB operation)) OR ((TI operations) OR (AB operations)) OR ((TI operative) OR (AB operative)) OR ((TI procedure) OR (AB procedure)) OR ((TI procedures) OR (AB procedures)) OR ((TI surgery) OR (AB surgery)) OR ((TI surgeries) OR (AB surgeries)) OR ((TI surgical) OR (AB surgical)) OR ((TI preoperative) OR (AB preoperative)) OR ((TI intraoperative) OR (AB intraoperative)) OR ((TI postoperative) OR (AB postoperative)) OR ((TI perioperative) OR (AB perioperative))) with limiters of a published date from 1998/01/01
	ScienceDirect search strategy
December 14, 2016	title-abs-key(metabolic syndrome) OR title-abs-key(syndrome x) OR title-abs-key(deadly quartet) OR title-abs-key(metsy) OR title-abs-key(metsyn) AND title-abs-key(intervention*) OR title-abs-key(operati*) or title-abs-key(procedure*) OR title-abs-key(surg*) OR title-abs-key(preoperative) OR title-abs-key(intraoperative) OR title-abs-key(postoperative) OR title-abs-key(perioperative) AND LIMIT-TO (yearnav, “2017, 2016, 2015, 2014, 2013, 2012, 2011, 2010, 2009, 2008, 2007, 2006, 2005, 2004, 2003, 2002, 2001, 2000, 1999, 1998”) AND LIMIT-TO (contenttype, “JL, BS”, “Journal”).
	EMBASE search strategy
December 14, 2016	‘metabolic syndrome’:ab,ti OR (syndrome:ab,ti AND x:ab,ti) OR (deadly:ab,ti AND quartet:ab,ti) AND surgery:ab,ti AND [1998-2016]/py AND [english]/lim AND [humans]/lim

## Additional file

Additional file 1: PRISMA-P 2015 checklist. (DOCX 73 kb)

## Abbreviations

CABG: Coronary artery bypass graft
CINAHL: Cumulative Index to Nursing and Allied Health Literature
MetS: Metabolic syndrome
MOOSE: Meta-analysis of observational studies in epidemiology (MOOSE) guidelines
PRISMA: Preferred Reporting Items for Systematic Reviews and Meta-Analyses
TJA: Total joint arthroplasty
